# What may happen when language teacher emotions and language policy intersect?

**DOI:** 10.3389/fpsyg.2022.935883

**Published:** 2022-07-18

**Authors:** Xiaohong Hu, Xinmin Zheng

**Affiliations:** ^1^School of English Studies, Shanghai International Studies University, Shanghai, China; ^2^School of Education, Shanghai International Studies University, Shanghai, China

**Keywords:** feeling rules, emotion labor, emotional capital, language policy, California

## Introduction

Served as both knowledge transmitters and student educators, teachers seem to have the upper hand in classrooms. However, this may not be the case beyond classrooms. Though esteemed as authorities and professionals in teaching, teachers are nothing but employees of institutions *per se*. The transformation in role makes it hard for teachers, subordinated and disadvantaged in power relations, to effectuate their professional beliefs and consequently conflicting emotions start to emerge. This is very true especially when a new policy is promulgated and teachers are obliged to meet corresponding requirements and expectations. These conflicting emotions, as we see it, deserve more attention and further investigation for they have, for a long time, been neglected by researchers and remained largely unknown to the public (Benesch, 2017).

With academic interest and practical concerns, we read the newly-published article Her and De Costa (2022) which focused on how a language teacher belonging to rather disfavored groups in power relations dealt emotionally with policy demands. To be more specific, the article took a critical poststructural perspective to investigate how a part-time non-native English-speaking teacher (NNEST) utilized the emotion labor that resulted from the feeling rules accompanied by a new language policy and ultimately accumulated emotional capital. As we see it, the article is full of exploratory and thought-provoking results worthy of being taken into account by both researchers and practitioners in the future. Thus, we would like to share our analysis of the article and make critical comments on it as well, hoping to provide illuminating insights for future research and practice. For the sake of clarity and convenience, the article Her and De Costa (2022) we are about to comment on will be referred to as “the article” in particular.

Before we analyze the article in detail, we think it is necessary to explicate the origins of and modifications to the two key terms mentioned in the second paragraph, namely, feeling rules and emotion labor. The two terms, coined by Hochschild (1983, 2012), were originally used in corporate settings and not until recently brought to language teaching research. In corporate settings, feeling rules are deemed as tacit expectations about the normative and appropriate reactions to particular workplace situations. When there is a clash between tacit expectations and employees' professional training and beliefs, emotional labor emerges. In the field of language teaching, Benesch (2012, 2017) removed the negative connotation of “emotional” by replacing it with “emotion” and further reconceptualized emotion labor and feeling rules either by challenging or extending previous definitions given by Hochschild.

First and foremost, Benesch refuses to accept that emotion labor is simply negative and individualized in nature (Hochschild, 1983). She considers emotion labor not as an indicator of teachers' emotional illiteracy, but as a healthy sign that institutional reform may be needed instead (Benesch, 2018). She also believes that emotion labor is socially- and discursively constructed, which implies that emotion labor is shaped by sociopolitical factors, such as power relations. Moreover, she discovers that feeling rules are not always explicit, especially in educational settings. It means that the onus is on researchers to deduce and analyze implicit feeling rules of educational policies before delving into how emotion labor is generated and dealt with.

Guided by such reconceptualization of feeling rules and emotion labor, Benesch (2018) proposed a poststructural-discursive approach to examine the relationship between emotions and power. This recently-proposed approach underscores that emotions are socioculturally regulated and discursively constructed, rather than manifested in individuals psychologically (Benesch, 2012, 2017). To date, Benesch has applied the poststructural-discursive approach to emotions to investigate a wide range of institutional policies, such as a plagiarism policy (Benesch, 2018), an attendance policy (Benesch, 2019) and a policy on high-stakes literacy testing (Benesch, 2020). All these attempts made by Benesch attest to and further enhance the applicability of a poststructural-discursive approach to language teacher emotions.

Mainly based on Benesch's (2018) research findings, the article adopted a critical poststructural approach to investigate what kind of feeling rules a new language policy implicitly demanded and how a part-time language teacher's emotion labor was generated and finally transformed into emotional capital.

## The study

By adopting a critical lens to language teacher emotions and language policy, the article can be seen as a timely response to growing calls to examine language teacher emotions critically (Gkonou and Miller, 2021) and to explore the interface between SLA and language policy (Han et al., 2019). Additionally, by borrowing the concept of emotional capital (Zembylas, 2007) to better understand the agentive potential of emotion labor, the article can also be regarded as a constituent of the very few research studies that attempt to unveil the accumulating process of emotional capital from emotion labor (Gkonou and Miller, 2021). Taken together, we consider that the article is of great value as to some extent it has overcome the afore-mentioned inadequacy in research on emotions and power and on emotional capital accumulated from emotion labor.

Qualitative in nature, a case study design was employed in the article. According to Duff (2020), it is essential to clarify researcher reflexivity and diversify data sources when undertaking a case study. As expected, a separate paragraph was written to unpack the researchers' reflections on their own positionality in research and multi-faceted data were gathered from diversified sources: a short background questionnaire, interviews, journal prompts, observations and policy documents. Even though the bulk of data came from interviews, we still highly appreciate the researchers' awareness of carrying out classroom observations in attempting to demystify emotions in practice. If there were any improvements to the research design, it would be that more observational data should be collected and analyzed since emotions in practice remains largely vague and elusive. Just as the researchers themselves advocated, future research could trace the relationship between policy negotiation and actual classroom practice in order to capture what is really going on in teachers' emotional practice and language policy implementation.

Bearing a critical poststructural perspective in mind, we will further illustrate the intersection between language teacher emotions and language policy by digging deeper into Alan's case investigated in the article. In Alan's case, the implicit feeling rules were deduced as trusting in the new language policy, the institution and the students. In other words, Alan was supposed to comply with the new language policy and have faith in students' abilities to conduct self-guided assessment. However, this expected trusting collided with Alan's professional training and beliefs and thereby conflicting emotions were generated. Although Benesch (2018) advocated for resistance to feeling rules from a university plagiarism policy, it might not be a universal solution to handle the generated emotion labor because not all teachers share the same identity that makes them least worry about their career development (Her and De Costa, 2022). Working as a part-time language teacher, Alan was reluctant to resist the feeling rules as he was afraid of losing his career. Since emotions are defined as feelings that lead to actions through the critical poststructural lens, it is reasonably assumed that emotion labor can be managed to trigger positive actions to improve the situation. When Alan was stuck in the whirlpool of conflicting emotions, the agentive potential of his emotion labor started to come into play. Instead of resisting the feeling rules boldly or succumbing himself to the mercy of emotion labor negatively, Alan developed his Christian spirituality and empathy as strategies to cultivate a more positive disposition and successfully accrued emotional capital. The accumulated emotional capital, consequently, was of great assistance to Alan in weathering the challenges at work and enabling him to tackle similar situations in the future. In short, the whole accumulating process of emotional capital proves that the agentive potential of emotion labor can be achieved through executing crafted coping strategies.

Obviously, the article is of great significance in overcoming research inadequacy, conducting an exemplary case study and reporting informative research findings. Nevertheless, we believe that the research design and the writing of the article could be improved, as illustrated next.

## Discussion

On the whole, the article leaves us with an impression that seemingly new language policies always run against language teachers' professional training and beliefs. This rough impression is reinforced when we read the sentence “Instead of being weighed down by the pessimism that pervaded the colleagues in his department, he chose to embrace optimism for the future” [(Her and De Costa, 2022), p. 7]. This sentence implies that the majority of teachers in Alan's department were pessimistic about the new language policy. In fact, we are quite skeptical about this statement because we find no survey or questionnaire results in the article to prove that. In our opinion, it would be more appropriate for the researchers to conduct a survey or distribute questionnaires to figure out language teachers' authentic attitudes and emotions toward the new language policy before jumping to conclusions.

Furthermore, the limited number of participants and the emphasis on Christianity may affect the generalizability and applicability of the research findings. We suggest future researchers to increase the number of participants by including other staff members such as full-time language teachers and department chairs, as advocated by the researchers themselves. On the other hand, Christianity as a strategy to address emotion labor may not be equally applied to generally irreligious countries such as China. We therefore expect more research studies to be conducted in diverse contexts, especially in China, so as to gain a more holistic understanding of language teachers' emotion labor.

In addition, some of the wording and phrasing in the article is worth discussing. Firstly, Alan's Christian faith was regarded as a positive emotion in the footnote, but later it was labeled as a neutral feeling in body paragraphs [see (Her and De Costa, 2022), p. 3, 4]. Secondly, it was clearly stated that four interviews were carried out, but the expression “in the fourth and final interview” in page seven was quite confusing. Thirdly, we believe that it was a mistake made by the researchers to type “emotional capital” as “emotion capital.” Even though this mistake only occurred once, we still feel obliged to point it out here for it may mislead readers. Given that “emotional labor” and “emotion labor” differ in connotations, readers may wonder whether there are any differences between “emotional capital” and “emotion capital.” In short, to avoid the afore-listed confusing situations, more attention should be paid to the consistency and unambiguity of the wording and phrasing when reporting research.

Last but not least, the unpleasant fact unraveled by the researchers is rather suggestive and illuminating for both institutional administrators and language teachers, that is, “emotion management can only be good because the institution frames it as good” [(Her and De Costa, 2022), p. 8]. For institutional administrators, it is anticipated de facto that language teachers are subjected to emotion labor generated from feeling rules accompanied by new language policies. For language teachers, they have no choice but to manage their emotions due to their disadvantaged positions in power relations. In other words, language teachers' self-management of emotions is actually welcomed and advocated by institutional administrators, for the reason that it is conducive to promote the implementation of language policies. However, emotions, if not managed well, may bring about attrition and burnout (Wolff and De Costa, 2017). To prevent this fallout, we advise institutional administrators to regard emotion labor as a healthy sign that institutional reform may be needed (Benesch, 2018), take steps to provide support for language teachers, and revise outdated or misguided language policies if necessary.

## Conclusion

To sum up, we believe that both researchers and practitioners can develop a more critical, comprehensive and sophisticated understanding of language teacher emotions and how it intersects with language policy after reading the article. Hence, we have no hesitation in recommending the article to larger reader groups who are particularly interested or directly involved in the whirlpool of emotion labor triggered by language policy, especially a new one.

## Author contributions

XH drafted the opinion. XZ helped XH to select the commented article, provided insights and suggestions during her writing, and helped revise the text. Both authors contributed to the article and approved the submitted version.

## Conflict of interest

The authors declare that the research was conducted in the absence of any commercial or financial relationships that could be construed as a potential conflict of interest.

## Publisher's note

All claims expressed in this article are solely those of the authors and do not necessarily represent those of their affiliated organizations, or those of the publisher, the editors and the reviewers. Any product that may be evaluated in this article, or claim that may be made by its manufacturer, is not guaranteed or endorsed by the publisher.

## References

[B1] Benesch S. (2012). Considering Emotions in Critical English Language Teaching: Theories and Praxis. New York: Routledge.

[B2] Benesch S. (2017). Emotions and English Language Teaching: Exploring Teachers' Emotion Labor. New York: Routledge.

[B3] Benesch S. (2018). Emotions as agency: Feeling rules, emotion labor, and English language teachers' decision-making. System. 79, 60–69. 10.1016/j.system.2018.03.015

[B4] Benesch S. (2019). “Feeling Rules and Emotion Labor: A Poststructural-Discursive Approach to English Language Teachers' Emotions,” in Second Handbook of English Language Teaching, eds Gao, X., Springer International Handbooks of Education. (Cham: Springer).

[B5] Benesch S. (2020). Emotions and activism: English language teachers' emotion labor as responses to institutional power. Crit. Inq. Lang. Stud. 17, 26–41. 10.1080/15427587.2020.1716194

[B6] Duff P. (2020). “Case study research: making language learning complexities visible,” in The Routledge Handbook of Research Methods in Applied Linguistics, eds McKinley, J., and Rose, H., (New York, Oxon: Routledge). 144–153.

[B7] Gkonou C. Miller E. (2021). An exploration of language teacher reflection, emotion labor and emotional capital. TESOL Q. 55, 134–155. 10.1002/tesq.580

[B8] Han Y. De Costa P. I. Cui Y. (2019). Exploring the language policy and planning/SLA interface: Ecological insights from an Uyghur youth in China. Lang. Policy. 18, 65–86. 10.1007/s10993-018-9463-9

[B9] Her L. De Costa P. I. (2022). When language teacher emotions and language policy intersect: a critical perspective. System. 105, 102745. 10.1016/j.system.2022.102745

[B10] Hochschild A. R. (1983). The Managed Heart: Commercialization of Human Feelings. Berkeley, CA: University of California Press.

[B11] Hochschild A. R. (2012). The Managed Heart: Commercialization of Human Feeling, Updated With a New Preface. Berkeley: University of California Press.

[B12] Wolff D. De Costa P. I. (2017). Expanding the language teacher identity landscape: an investigation of the emotions and strategies of a NNEST. Mod. Lang. J. 101, 76–90. 10.1111/modl.12370

[B13] Zembylas M. (2007). Emotional capital and education: theoretical insights from Bourdieu. Br. J. Educ. Stud. 55, 443–463. 10.1111/j.1467-8527.2007.00390.x

